# Aging in the Workplace: The Role of the ADEA, ADA, and AI in Shaping the Future of Work

**DOI:** 10.1093/geroni/igaf122.1817

**Published:** 2025-12-31

**Authors:** Rima Nathan

**Affiliations:** Florida State University, Tallahassee, Florida, United States

## Abstract

Despite the protections of the Age Discrimination in Employment Act (ADEA) and the Americans with Disabilities Act (ADA), age discrimination remains deeply embedded in workplace structures. While these laws were designed to prevent bias, they often fall short due to weak enforcement and the intersectional nature of workplace discrimination. Older workers—especially women—face unique challenges, including compounded bias based on gender and caregiving responsibilities, disparities in promotions and pay, and systemic barriers to reemployment after workforce exits. This presentation will examine how the legal framework meant to protect older workers sometimes reinforces inequities instead. It will explore the disproportionate impact on women in mid-to-late career stages and the challenges for all of proving age bias under current law. Additionally, with the rise of artificial intelligence in hiring and employment decisions, new forms of age-based exclusion are emerging, further complicating legal and policy responses. Through a critical analysis of these issues, this presentation will highlight areas for policy reform, stronger enforcement mechanisms, and employer best practices to create truly inclusive workplaces. Attendees will gain a deeper understanding of how legal structures, workplace culture, and evolving technologies intersect to shape the experiences of older workers—and what can be done to dismantle these barriers.

